# Intermittent hypoxia increases kidney tumor vascularization in a murine model of sleep apnea

**DOI:** 10.1371/journal.pone.0179444

**Published:** 2017-06-08

**Authors:** Antoni Vilaseca, Noelia Campillo, Marta Torres, Mireia Musquera, David Gozal, Josep M. Montserrat, Antonio Alcaraz, Karim A. Touijer, Ramon Farré, Isaac Almendros

**Affiliations:** 1 Unitat de Biofísica i Bioenginyeria, Facultat de Medicina i Ciències de la Salut, Universitat de Barcelona, Barcelona, Spain; 2 Urology Department, Hospital Clínic de Barcelona, Barcelona, Spain; 3 Institute for Bioengineering of Catalonia, Barcelona, Spain; 4 Centro de Investigación Biomédica en Red de Enfermedades Respiratorias, Madrid, Spain; 5 Laboratori del son, Servei de Pneumologia, Hospital Clínic, Barcelona, Spain; 6 Department of Pediatrics, Pritzker School of Medicine, Biological Sciences Division, The University of Chicago, Chicago, United States of America; 7 Institut d'Investigacions Biomèdiques August Pi i Sunyer, Barcelona, Spain; 8 Urology Service, Department of Surgery, Memorial Sloan Kettering Cancer Center, New York, NY, United States of America; Centro Cardiologico Monzino, ITALY

## Abstract

We investigate the effects of intermittent hypoxia (IH), a characteristic feature of obstructive sleep apnea (OSA), on renal cancer progression in an animal and cell model. An *in vivo* mouse model (Balb/c, n = 50) of kidney cancer was used to assess the effect of IH on tumor growth, metastatic capacity, angiogenesis and tumor immune response. An *in vitro* model tested the effect of IH on RENCA cells, macrophages and endothelial cells. Tumor growth, metastatic capacity, circulating vascular endothelial growth factor (VEGF) and content of endothelial cells, tumor associated macrophages and their phenotype were assessed in the tumor. *In vitro*, VEGF cell expression was quantified.Although IH did not boost tumor growth, it significantly increased endothelial cells (p = 0.001) and circulating VEGF (p<0.001) in the *in vivo* model. Macrophages exposed to IH *in vitro* increased VEGF expression, whereas RENCA cells and endothelial cells did not. These findings are in keeping with previous clinical data suggesting that OSA has no effect on kidney cancer size and that the association observed between OSA and higher Fuhrman grade of renal cell carcinoma may be mediated though a proangiogenic process, with a key role of macrophages.

## Introduction

Obstructive sleep apnea (OSA) is a highly prevalent sleep disorder among adult population and is characterized by recurrent occlusions of the upper airway during sleep hours leading to intermittent hypoxia (IH) [1]. The most common risk factors of OSA are age, male gender and obesity [2]. Patients suffering from OSA present increased levels of oxidative stress, systemic inflammation and hypoxia-inducible factors (HIF) including their downstream gene products such as vascular endothelial growth factor (VEGF), a regulator and promoter of angiogenesis [3,4]. A growing body of evidence based on large observational population-based studies supports a link between OSA and an increase in cancer incidence and mortality [5–7]. For kidney cancer in particular, a higher incidence among patients with OSA has been recently reported [7], and an association between OSA and high Fuhrman grade among patients undergoing surgery for clear cell renal cell carcinoma (ccRCC) has been described [8]. It has been hypothesised that IH in early cancers may select for specific genotypic and phenotypic properties that persist even in normoxic conditions and may promote tumor progression and resistance to therapy [9].

Better understanding of the pathophysiology of RCC in recent years has shown the important role of hypoxia through HIF-1α pathways [10–13], which has led to successful application of antiangiogenic drugs targeting VEGF [14]. Thus, HIF-1α and its downregulated genes have an important role in the pathophysiology of both OSA and RCC. In fact, increased levels of VEGF in response to IH have been previously described in a mouse melanoma model [15] and clinically in patients with OSA [4,16].

The immune system is now considered to play a key role in tumor angiogenesis, invasion, intravasation and metastasis [17]. In particular, tumor associated macrophages (TAMs) have been reported as potentially involved in tumor growth. TAMs can have two different phenotypes (M1 and M2). While M1 phenotype is associated to tumor inhibition, M2 phenotype is related to tumor promotion [18]. In an animal model of lung cancer, mice undergoing IH presented an accelerated TAMs transition from M1 to M2 phenotype [19].

Our primary hypothesis here is that IH promotes the synthesis of proangiogenic factors such as VEGF, enhancing the neovascularization of the RCC tumor. Secondarily, we hypothesized that in RCC, IH promotes a shift toward M2 phenotype in macrophages thereby triggering the proliferation of tumor cells, similar to what was observed in lung carcinoma [19]. Additionally, we aimed to investigate the potential sources of VEGF in response to IH from different cell types involved in kidney cancer pathogenesis and known to produce VEGF (RCC cells, macrophages and endothelial cells) [20,21]. Altogether, this study aims to provide biological plausibility of those findings recently observed in OSA patients with ccRCC undergoing surgery [8].

## Materials and methods

### Animals and cell lines

The study was approved by the Comitè Etic d'Experimentació Animal (CEEA) of the Universitat de Barcelona (Permit number 192/15). All surgery was done under isoflurane anesthesia, and all efforts were made to minimize suffering. The study was conducted on pathogen-free, 8-week-old Balb/c male mice (Charles River Laboratories, Saint Germain sur L’arbresle, France). All animals were housed in controlled cages and were fed with standard rodent chow (Panlab, Barcelona, Spain) and tap water *ad libitum*. They were all acclimated for one week prior to the experiments and kept with 12:12 h light-dark cycle. All surgical procedures were done under general anesthesia with intraperitoneal injection of a ketamine and xylazine combination (100mg/Kg; 10mg/Kg). Animals were monitored every 5 minutes throughout the experimental procedure by observing that there was no change in respiratory rate associated with surgical manipulation and toe pinch. Analgesia was procured with subcutaneous injection of buprenorphine 0.05mg/Kg before the procedure and after it every 12 h during 72 h. Wet food was put inside the cage during the first 24 h after the surgical procedure to facilitate feeding. Immediately prior to euthanasia, isoflurane was mixed with mineral oil at a concentration of 20% and placed inside the induction chamber. The maximum tumor size permitted during the experiment was 1.2 cm in diameter.

A well stablished murine model of kidney adenocarcinoma based on RENCA cells (American Type Culture Collection (ATCC)-CRL-2947, Manassas, VA, USA) was used for *in vivo* and *in vitro* experiments [22]. Kidney cancer cells were cultured in RPMI 1640 medium (ATCC, 30–2001) supplemented with 10% fetal bovine serum (FBS) (Gibco 10270, Thermo Fisher Scientific, Waltham, MA, USA), 100 U/ml penicillin and 100 μg/ml streptomycin (Sigma-Aldrich, Missouri, USA). To assess the potential source of VEGF *in vitro*, mouse macrophages RAW 264.7 and human primary aortic endothelial cells (HAEC) were also employed in addition to RENCA cells. RAW 264.7 and HAEC cells were obtained from ATCC (TIB-71 and PCS-100-011, respectively). RAW 264.7 cells were cultured in high glucose Dulbecco’s modified Eagle’s medium (DMEM) supplemented with 10% FBS, 100 U/ml penicillin, 100 μg/ml streptomycin and 0.250 μg/ml amphotericin B and maintained at 37°C and 5% CO_2_. In other hand, HAEC cells were routinely grown in vascular cell basal medium supplemented with endothelial cell growth kit-VEGF (ATCC) supplemented with an antibiotic/antimycotic solution at final concentrations of 10 U/ml penicillin, 10 μg/ml streptomycin and 25 μg/ml amphotericin B. All cell cultures were maintained in T-25 tissue culture flasks in a standard humidified incubator at 20% O_2_, 5% CO_2_ and 37°C prior to experimentation.

### Application of intermittent hypoxia in vivo

The experimental setting for applying IH mimicking OSA in mice has been previously described by our group [15]. The IH group was submitted to a continuous flow of gas cyclically switched from room air (40 s) to a gas reservoir of hypoxic air at an oxygen fraction of 5% (20 s). The control group was identically instrumented but received room air (RA) all the time. Animals were pre-exposed for two weeks to RA or IH prior to the subcutaneous or orthotopic RCC model induction as detailed below.

### Subcutaneous kidney cancer model: Assessment of tumor growth, cell proliferation, angiogenesis, circulating VEGF levels and macrophage polarization

A subcutaneous kidney tumor was induced into the right flank region in twenty-two mice (11 mice per group) injecting 2x10^5^ RENCA cancer cells suspended in 100 μl of PBS subcutaneously. After twelve days, tumors were palpable and visible in all mice. After 21 days from tumor induction, mice were anesthetized, exsanguinated and euthanized by cervical dislocation. The tumors were immediately excised and weighted. Samples for immunohistochemistry (IHC) and flow cytometry analysis were stored. Plasma samples were used to measure circulating VEGF by ELISA (Quantikine, R&D Systems, Minenapolis, USA), following the manufacturer’s protocol.

To carry out flow cytometry, seven tumor samples from each group were mechanically disrupted in small pieces and digested in collagenase 1 mg/mL for 1 h in the shaker at 37°C. Cells were harvested and filtered through a 100 μM nylon mesh cell strainer (352350, BD Falcon, Bedford, US). After washing the cells three times they were incubated for 30 minutes at 4°C and stained with surface markers antibodies. Endothelial cells were identified as CD31+ cells and tumor associated macrophages (TAMs) as CD45+,CD11b+,F4/80+ cells. To evaluate cell phenotype, CD206 and CD86 were added to the macrophage panel as M2 and M1 markers, respectively. All antibodies were purchased from Biolegend (San Diego, US). Finally, the percentage of each cell type was quantified to assess the vascularization of the tumor and the content of macrophages and their polarization (M1 and M2).

The rest of the samples from each group were fixed in formalin and embedded in paraffin to stain the blood vessels (CD31+ cells) and quantify the proliferation rate (Ki-67 marker). The paraffin blocks of the tumors were cut into 5 μm sections, dewaxed, rehydrated and prepared using heat-induced epitope retrieval in citrate buffer, pH 6.0. Anti-CD31 (ab28364, Abcam, Cambridge, UK) and anti-Ki67 (ab15580, Abcam, Cambridge, UK) rabbit polyclonal antibodies were used following the manufacturer’s protocol. Sections were finally counterstained with hematoxylin. Quantitative analysis was done using ImageJ (Wayne Rasband). Six fields from each sample avoiding necrotic areas at x20 magnification for CD31+ and x40 magnification for the Ki67 were used for the analysis. The selection of the fields and posterior analysis were performed in blinded conditions.

### Orthotopic kidney cancer model: Assessment of metastatic capacity

Twenty-eight mice were used to assess the metastatic capacity of IH. Animals were identically randomized and subjected to either RA or IH as previously described. To generate this model, the mice were anesthetized using 2–4% isoflurane (Baxter, Deerfield, US). The incision area and its surroundings were shaved and cleaned with 70% ethanol and chlorhexidine scrub. A 5–10 mm incision was done in the outer skin layer of the right flank to expose the interior muscle layer, which was then opened to expose the right kidney. The kidney was exteriorized gently and the cell suspension (1x10^5^ RENCA cells suspended in 20 μl of PBS) was injected into the subcapsular space. Once the kidney was replaced to its original position, the interior wall was closed with a Vicryl 3/0 (Ethicon, Somerville, US) and the skin with polypropylene 2/0 suture (Aragó, Barcelona, Spain). Subcutaneous buprenorphine (Buprex, RB Pharmaceuticals Limited, UK) at a concentration of 0,05 mg/Kg was administered before the incision and every 12 h for three days after the procedure. The skin suture was removed seven days after the procedure.

After 20 days of tumor induction, mice were exsanguinated and sacrificed by cervical dislocation under anesthesia. The lungs were then harvested, fixed in paraformaldehyde and embedded in paraffin. Paraffin blocks were sectioned into 5 μm sections, stained with hematoxilyn/eosin and examined under light microscopy by a double-blind investigator. Total number of metastasis and metastatic area was quantified using Metamorph image analysis software (Molecular Devices, Sunnyvale, CA, USA).

### Assessment of VEGF production in RENCA cells, macrophages and endothelial cells exposed to IH in vitro

To assess the potential sources of VEGF under IH conditions, 5x10^4^ RENCA cells, 15x10^4^ RAW 264.7 cells and 6x10^4^ HAEC cells were exposed to IH (cycles alternating 20% O_2_−1% O_2_ every 30 s) or continuous RA (21% O_2_) as previously described [23]. After 24 h, total RNA from RENCA, RAW 264.7 and HAEC cell cultures were extracted using the RNeasy kit (Qiagen, Hilden, Germany). One μg was employed to synthesize cDNA by reverse transcription polymerase chain reaction (TaqMan Reverse Transcription Reagents) according to manufacturers’ instructions. qPCR was performed using the Taqman Fast Advanced Master Mix and the TaqMan Gene Expression Assays in a StepOnePlus thermocycler. Relative VEGF gene expression normalized to eukaryotic 18S rRNA endogenous control was calculated using the 2-ΔΔCT method [24] to quantify fold change in VEGF gene expression compared to baseline. All reagents were purchased from ThermoFisher Scientific (Waltham, MA) unless otherwise noted.

### Statistical analysis

All data are presented as mean ± SE. T-student test was used to compare IH and RA groups. Two-tailed P value of <0.05 was considered to achieve statistical significance. The Spearman Rank correlation test was used to evaluate the correlation between plasmatic VEGF and endothelial cells.

## Results

### Intermittent hypoxia induces tumor angiogenesis, macrophage recruitment and increases systemic VEGF levels in the subcutaneous model of RCC

**Fig 1:**
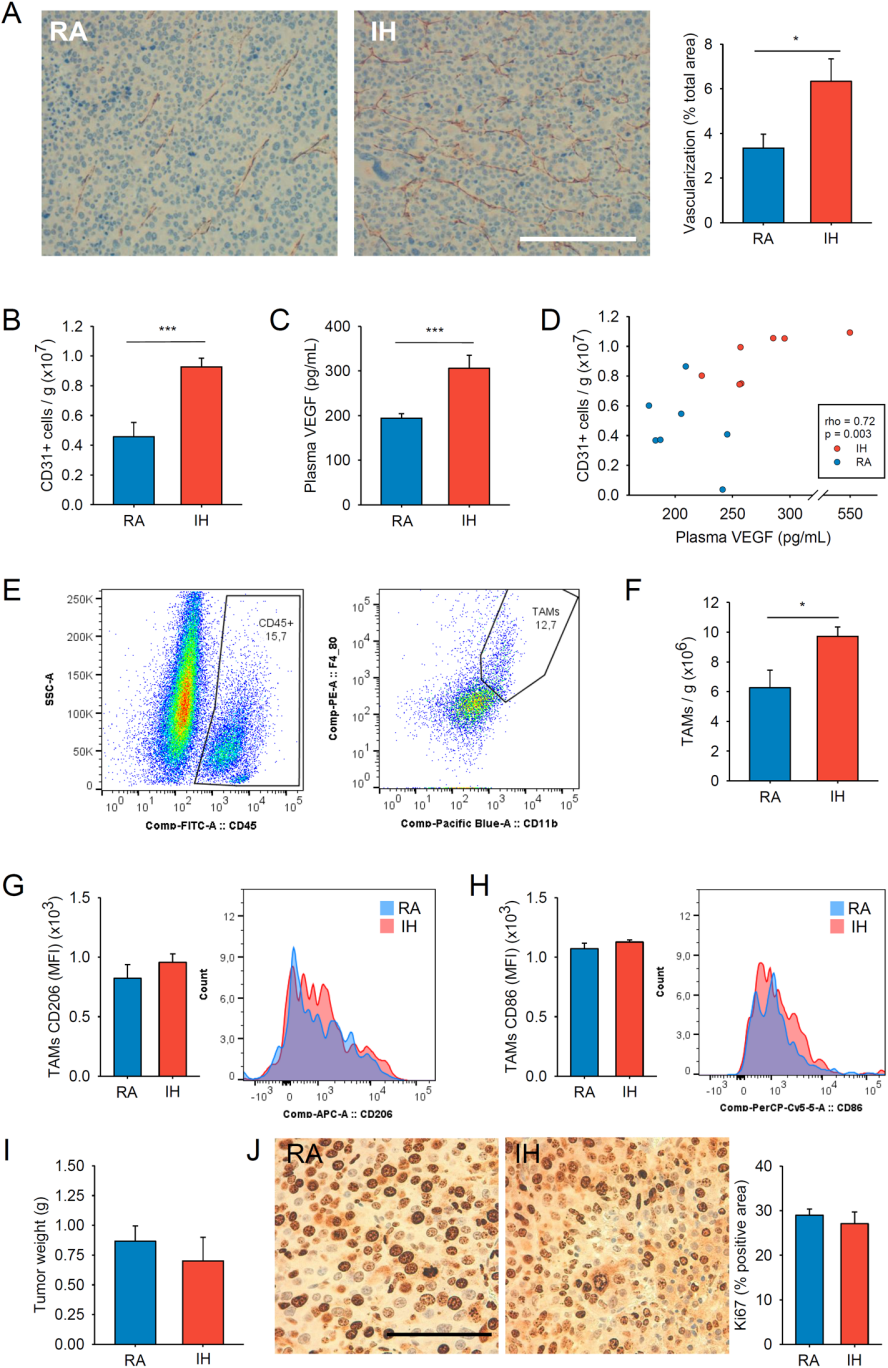
Results from the subcutaneous RCC model: A) Application of intermittent hypoxia (IH) markedly increased vascularization (stained in brown) compared to room air (RA) (scale bar 200 μm). B) Flow cytometry analysis revealed similar increase in the number of endothelial cells within the tumors of mice exposed to IH. C) Animals exposed to IH also presented increased circulating levels of VEGF which D) correlated with tumor vascularization. E) Tumor-associated macrophages (TAMs) were identified as CD45+, CD11b+ and F4/80+ cells by flow cytometry and F) their analysis revealed that IH promotes an increased cell density of TAMs in comparison to room air (RA) conditions. However, IH did not change any of the markers G) CD206 (pro-tumoral M2 marker) and H) CD86 (anti-tumoral M1 marker) studied. In both cases, representative histograms on their expression are shown for RA and IH conditions. Despite the increased tumor vascularization assessed under IH conditions, I) IH did not change tumor weight nor J) tumor cells proliferation measured by the immunohistochemical analysis of the Ki67 expression. Representative images of Ki67 staining obtained from mice exposed to RA and IH are shown (scale bar 100 μm). Data is presented as mean ± SE. * p<0.05 and *** p<0.001.

At the end of the study, tumor sections revealed a 1.9-fold increase (p = 0.044) in the density of vessels in those mice exposed to IH respect to RA (Fig 1A). The higher vessel density in tumors of mice exposed to IH was also supported by the flow cytometry analysis. In particular, cell density of vascular endothelial cells within the tumor stroma similarly experienced a ~2-fold increase compared to the control group (p = 0.001) (Fig 1B). Plasma VEGF was significantly overexpressed in the IH group, with a mean concentration of 306±93 pg/mL compared to 194±10 pg/mL in the normoxic control group (p<0.001) (Fig 1C). Interestingly, a positive significant correlation was seen between circulating VEGF and endothelial cells (Rho = 0.42, p = 0.003) (Fig 1D). In addition, the application of IH increased the density of TAMs (cells per gram of tissue) on RENCA tumors. Specifically, TAMs density within the tumor under IH was increased by ~58% compared to normoxia (p = 0.024) (Fig 1E and 1F). However, we did not observe any change in the expression of the M1 (Fig 1G) and M2 (Fig 1H) surface markers of TAMs evaluated from IH-exposed RENCA tumors. No differences on tumor weight (Fig 1I) or proliferation rates (Fig 1J) were either observed.

### Intermittent hypoxia do not promote metastatic capacity in the orthotopic model of RCC

**Fig 2:**
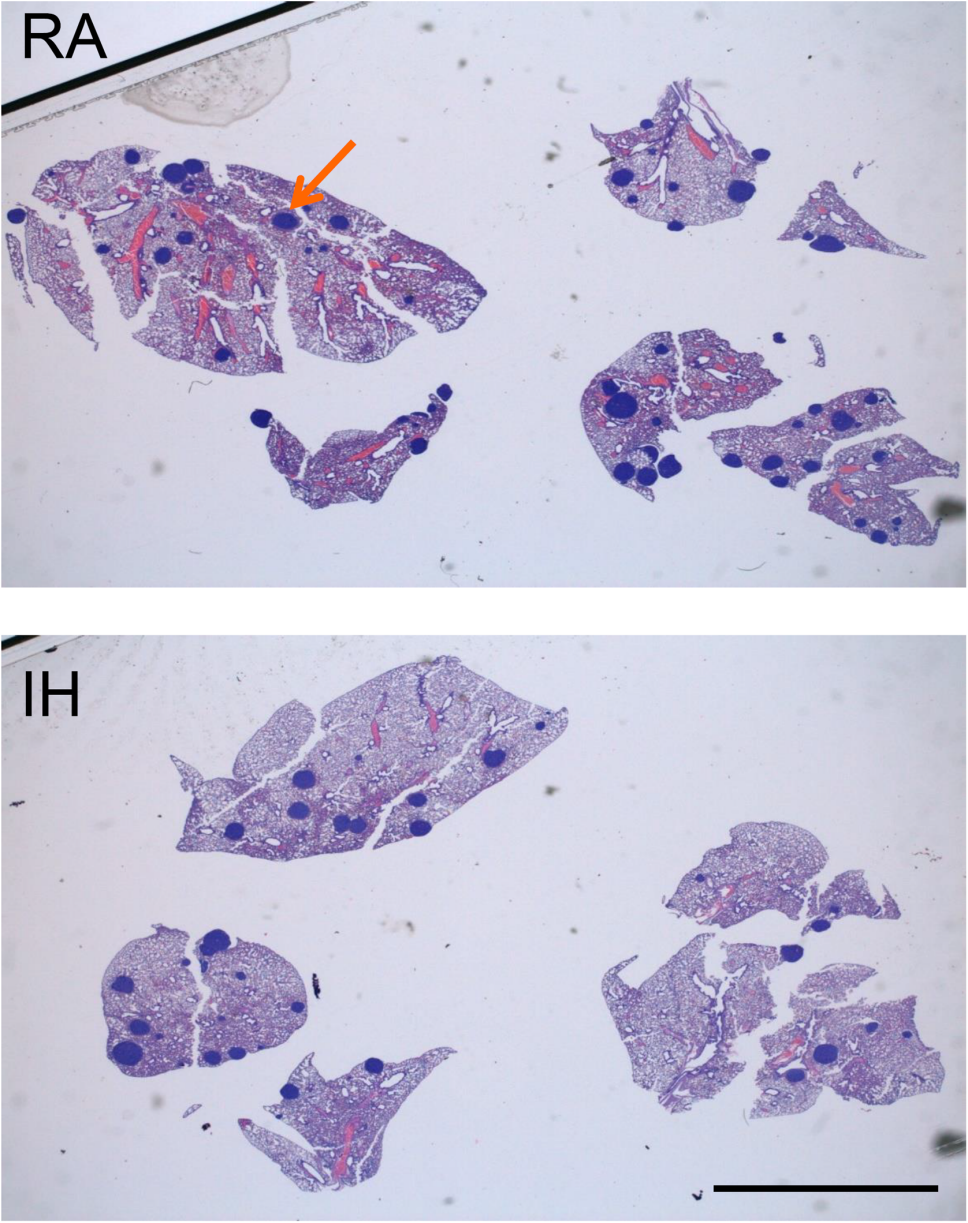
Results from the orthotopic RCC model: No differences in the metastatic capacity of RCC tumors were encountered between intermittent hypoxia (IH) and room air (RA) groups. The orange arrow points one of the observed lung metastasis (stained in blue). The images represent different lung lobes from the same animal exposed to either RA (top) or IH (bottom) (scale bar 5 mm).

In the orthotopic model, performed to assess metastatic capacity, no differences on lung metastases were observed between the study groups (Fig 2A). Specifically, the percentage of lung metastatic area was 0.29±1.61% and 0.90±0.57% (median±SE, p = 0.446) in mice exposed to RA and IH, respectively.

### IH promotes VEGF production by macrophages in vitro

**Fig 3:**
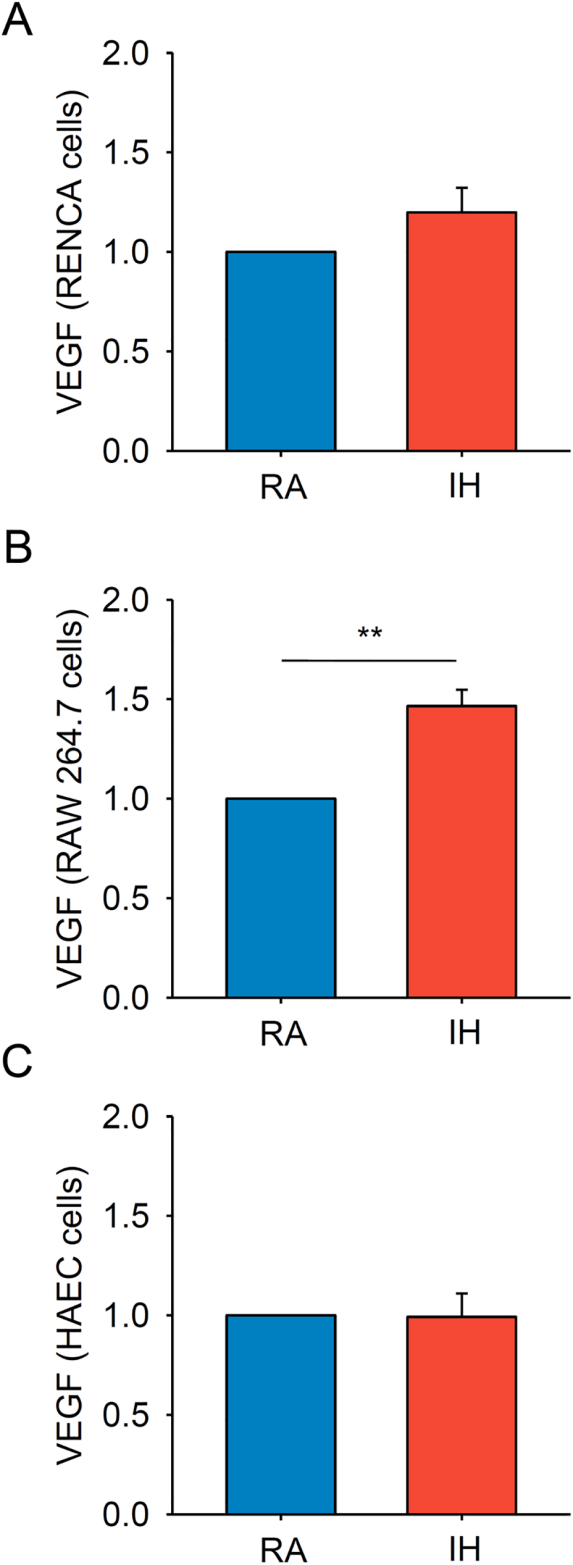
VEGF relative gene expression measured in A) RENCA, B) macrophages and C) endothelial cells showed a differential response to IH *in vitro*. Only macrophages presented a marked increase in VEGF expression in response to IH. Data is presented as mean ± SE. ** p<0.01.

The application of IH *in vitro* revealed a different response in tumor cells (Fig 3A), macrophages (Fig 3B) and endothelial cells (Fig 3C). Specifically, VEGF expression of RENCA cells exposed to IH for 24 h experienced a trend towards increased levels but the differences did not reach statistical significance. On the other hand, IH promoted a ~50% increase in VEGF expression in RAW 264.7 macrophages (p = 0.002) and no differences were observed in HAEC cells.

## Discussion

Application of IH mimicking OSA in our mouse model of kidney cancer markedly enhanced angiogenesis, which is commonly considered as an important malignant prognostic factor in kidney cancer [25]. HIF-1α pathways seem to have a role in ccRCC [10–13] and in other cancers, with context dependent roles [26]. In our study, elevated systemic levels of VEGF was observed in response to IH, explaining the higher vessel formation assessed by flow cytometry and histologically by IHC (Fig 1). In addition, the experiments carried out *in vitro* revealed the importance of the immune system on angiogenesis, supporting the role of macrophages on VEGF production in response to IH (Fig 3B).

In order to explore the potential relation between OSA and kidney cancer, we reviewed a large series of patients with ccRCC and found an increased risk of having a high Fuhrman grade in patients with OSA compared to those not diagnosed with OSA (51 vs. 38%; 13% risk difference; 95% confidence interval (CI), 5–20) [8]. In the same study, a VEGF pathway enrichment among OSA patients was suspected. However, there were no differences on tumor size, metastasis-free survival or cancer-specific survival. Interestingly, the mouse kidney cancer model used in the present study reproduced most of these clinical findings observed in humans, and no effect of IH on tumor growth (Fig 1I) and metastasis (Fig 2A) was seen.

IH has been associated with higher tumor growth [27], metastatic rates and mortality [28] compared to normoxic controls in melanoma and lung adenocarcinoma mouse models. Interestingly, this effect has not been reproduced in our RCC model. This fact could be explained in part by a differential immune response observed in the RENCA tumor model compared to previously reported findings [19]. In particular, lung adenocarcinoma cells are able to increase the recruitment of resident macrophages from surrounding tissues and bone-marrow derived monocytes and, most importantly, promote their shift towards a M2 phenotype under IH conditions [19]. In fact, *in vitro* assays previously demonstrated that the co-culture of TAMs isolated from tumors of mice exposed to IH is able to increase tumor cell proliferation, migration and invasion compared to TAMs isolated from the control group [19]. In our model of RENCA, we did not observe a shift toward a more pro-tumoral phenotype (M2) (Fig 1). Thus, the lack of IH-induced alterations on TAMs phenotype in our model could explain in part the absence of tumor growth differences between groups. Remarkably, our results are in accordance with the available data from the clinical study where no changes on ccRCC tumor growth or metastasis were observed in OSA patients. Further investigation is still needed to understand the differential response of the immune system in kidney cancer compared to other cancer types.

Microvessel density as a surrogate of angiogenesis has been described as an independent prognostic factor for metastasis in human ccRCC [25]. Also, plasmatic levels of VEGF are increased in patients with RCC and its level in serum correlates to clinical stage and histopathological grade [29]. From this perspective, plasma VEGF levels were significantly increased in mice subjected to IH, and had a local effect within the tumors, which were more vascularized than those in the control group. Also, we explored the potential sources of VEGF *in vitro* in order to investigate the potential contribution of tumor cells and other stromal cells such as macrophages or endothelial cells on tumor vascularization. After 24 h of IH exposure, macrophages showed a clear increase in VEGF expression. Conversely, RENCA cells only presented a trend towards increase in VEGF expression and no changes were observed in endothelial cells (Fig 3). These results suggest that the increased tumor vascularization observed under IH and explained by higher circulating VEGF levels is most likely due to an increased expression of VEGF by other cell populations in tumor stroma such as macrophages rather than from RENCA cells. Similar findings on VEGF levels and tumor vascularization were previously observed in mice subjected to IH in a melanoma cancer model [15].

The use of animal and *in vitro* models avoids the interaction of most common confounders such as obesity or other metabolic disorders associated with OSA, which are well known to promote cancer progression [30,31]. However, some limitations in our study should be mentioned. While our results reproduce most of the clinical findings from a patient based database [8], the involved molecular mechanisms should be tested in humans or in tumor models from patients [32]. Also, whereas we only studied the effect of IH, other noxious factors associated with OSA, such as sleep fragmentation, should be analyzed in the future [33].

## Conclusions

Our animal model results are consistent with previous clinical data showing that OSA does not modify RCC tumor size. Although the increased circulating levels of VEGF and tumor vascularization found in mice subjected to IH do not translate into changes in tumor growth and metastasis, they could participate in the increased Fuhrman grade observed clinically in OSA patients undergoing surgery for a RCC. However, further studies are still needed to fully understand the potential mechanisms that contribute to the increased malignancy observed clinically in patients with OSA. In the context of the relationship between OSA and cancer, the RCC model has a distinctive response to IH compared to other cancer models such as melanoma or lung adenocarcinoma, which highlights the importance of designing and performing new clinical studies based on specific types of cancers.

## Supporting information

S1 File Raw experimental data. (XLSX)
